# “At the mercy of some of the regulations”: the impact of the residency match and return of service requirement on the early-career decisions of international medical graduates in Canada

**DOI:** 10.1186/s12960-022-00709-0

**Published:** 2022-02-04

**Authors:** Maria Mathews, Dana Ryan, Ellen Randall, Emily Gard Marshall, Laurie J. Goldsmith, Lori Jones, M. Ruth Lavergne, David Snadden, Ian Scott, Sabrina T. Wong, Katherine Stringer, Kathleen Horrey, Agnes Grudniewicz

**Affiliations:** 1 Department of Family Medicine, Schulich School of Medicine & Dentistry, University of Western Ontario, 1151 Richmond Street, London, ON N6A 5C1 United Kingdom; 2 School of Population and Public Health, University of British Columbia, 2206 E Mall, Vancouver, BC V6T 1Z3 Canada; 3 Department of Family Medicine Primary Care Unit, Dalhousie University, 1465 Brenton Street, Suite 402, Halifax, NS B3J 3T4 Canada; 4 Faculty of Health Sciences, Simon Fraser University, 8888 University Drive, Burnaby, BC V5A 1S6 Canada; 5 Family Medicine and Primary Care, Lee Kong Chian School of Medicine, Nanyang Technological University, Clinical Sciences Building (Novena), 11 Mandalay Road, Singapore, 308232 Singapore; 6 Department of History, University of Ottawa, 55 Laurier Ave E, Ottawa, ON K1N 6N5 Canada; 7 Department of Family Medicine, Dalhousie University, 1465 Brenton Street, Suite 402, Halifax, NS B3J 3T4 Canada; 8 Department of Family Practice, University of British Columbia, Northern Medical Program, UNBC, 3333 University Way, Prince George, BC V2N 4Z9 Canada; 9 Department of Family Practice, University of British Columbia, 3rd Floor David Strangway Building, 5950 University Boulevard, Vancouver, BC V6T 1Z3 Canada; 10 UBC Centre for Health Services and Policy Research and School of Nursing, University of British Columbia, 201-2206 East Mall, Vancouver, BC V6T 1Z3 Canada; 11 Telfer School of Management, University of Ottawa, Desmarais Building, 55 Laurier Ave East, Ottawa, ON K1N 6N5 Canada

**Keywords:** Post-graduate medical education, Residency, International medical graduate, Early-career family physician, Return-of-service, Family medicine, Qualitative research

## Abstract

**Background:**

Return-of-service (ROS) agreements require international medical graduates (IMGs) who accept medical residency positions in Canada to practice in specified geographic areas following completion of training. However, few studies have examined how ROS agreements influence career decisions. We examined IMG resident and early-career family physicians’ perceptions of the residency matching process, ROS requirements, and how these factors shaped their early career decisions.

**Methods:**

As part of a larger project, we conducted semi-structured qualitative interviews with early-career family physicians and family medicine residents in British Columbia, Ontario and Nova Scotia. We asked participants about their actual or intended practice characteristics (e.g., payment model, practice location) and factors shaping actual or intended practice (e.g., personal/professional influences, training experiences, policy environments). Interviews were transcribed verbatim and a thematic analysis approach was employed to identify recurring patterns and themes.

**Results:**

For this study, we examined interview data from nine residents and 15 early-career physicians with ROS agreements. We identified three themes: IMGs strategically chose family medicine to increase the likelihood of obtaining a residency position; ROS agreements limited career choices; and ROS agreements delayed preferred practice choice (e.g., scope of practice and location) of an IMGs’ early-career practice.

**Conclusions:**

The obligatory nature of ROS agreements influences IMG early-career choices, as they necessitate strategically tailoring practice intentions towards available residency positions. Existing analyses of IMGs’ early-career practice choices neglect to distinguish between ROS and practice choices made independently of ROS requirements. Further research is needed to understand how ROS influences longer term practice patterns of IMGs in Canada.

## Background

In Canada, international medical graduates (IMGs) are physicians who, regardless of citizenship, graduated from medical school outside the country. IMGs face many hurdles qualifying for practice in Canada. Obtaining a post-graduate residency position in Canada is competitive [1]; in 2021, only 410 of 1356 IMG applicants matched to a residency position [2]. For those who do not have opportunities to complete residency training abroad, or whose post-graduate training or previous clinical experience is not recognized in Canada, residency is the only route that will qualify an IMG to work as a physician in this country. Unlike graduates of a Canadian medical school (CMGs), most residency positions available to IMGs in Canada are in family medicine and require trainees to fulfil return-of-service (ROS) agreements [3].

ROS agreements are used in many countries as a method of recruiting physicians with the intent to address physician shortages in rural or under-serviced communities [4–8]. In Canada, all provinces, with the exception of Alberta and Quebec, require the majority of IMGs who match to residency positions to complete ROS agreements, although the specific eligibility criteria and service terms vary (Table 1). Contract terms are tied to the residency matching process and vary by province, trainee type (IMGs or CMGs), the application round (or iteration), and whether the trainee has matched with a seat designated for another trainee type (e.g., IMG matching to a CMG-designated seat). IMG-designated positions are often limited to specific disciplines, most often family medicine. CMGs are also required to complete ROS agreements if they opt for specific training programs (e.g., Manitoba’s Northern Remote stream) or if they match to IMG-designated residency positions (e.g., in Prince Edward Island, New Brunswick, or Nova Scotia). Physicians who are unable or unwilling to complete their service commitment can avail of a repayment or “buy-out” option that requires them to pay back all, or a pro-rated portion, of the costs associated with their training, plus applicable interest, however, some provincial programs include additional penalties (e.g., British Columbia) or repayment requirements (e.g., Saskatchewan) to discourage IMGs from taking this option. Service commitments range from 1 to 5 years in length and are expected to begin upon completion of residency training. Notably, graduates of medical schools in the United States are considered equivalent to CMGs in the residency match, meaning they may apply at the same time as CMGs and compete for CMG-designated positions that do not require a ROS obligation [3]. Voluntary ROS programs are only available to CMG post-graduate trainees (Table 2). These ROS agreements differ from mandatory ROS requirements in that they provide CMGs with funding in the form of a bursary and are generally not a condition of residency admission. Voluntary ROS agreements are also available to CMG undergraduate trainees [6].

**Table 1:** Mandatory ROS requirements for post-graduate medical education trainees in Canada

Province	Program Name	Trainee Type		Round*	Program		Eligibility Criteria (CaRMS Stream**)	Time Requirement	Payback amount
		IMG	CMG		Family Medicine	Other			
BC	Return of Service Program [22]	✓		1	✓		Parallel stream [23]	2 years [24–25]	Cost of training + interest + estimated damages (cost of replacement physician + average annual MSP billings). [22]
		✓	✓	2			Competitive stream [23]		
SK	International Medical Graduate Postgraduate Medical Training [26]	✓		1,2	✓	✓	Competitive stream (first and second iteration for family medicine; first iteration only for other speciality) [23]	1 year ROS/1 year of training [27]	Costs of training + interest [27]
MB	No formal title	✓		1,2	✓	✓	Parallel stream [23]	1 year ROS/1 year of training [28]	Costs of training + interest paid in one lump sum [28]
ON	IMG Return of Service Program (IMG ROS) [29]	✓		1,2	✓	✓	Parallel stream; includes subspecialty positions [24]	5 years; physicians with multiple ROS adhere to most restrictive ROS [29]	Costs of training + interest + administrative costs; may be repaid in installments [29, 30]
NL	No formal title	✓		1	✓	✓	Parallel stream [23]	1 year ROS/1 year of training [24]	Prorated cost of training based on number months of ROS unfulfilled + interest [31–32]
		✓	✓	2			Competitive stream [23]		
NS	No formal title	✓		1	✓	✓	Graduates of Non-LCME/CACMS-Accredited Medical Schools (GNLGAMS) parallel stream [24]	1 year ROS/1 year of training [33–34]	Not specified
		✓	✓	2			GNLGAMS competitive stream [24]		
NB	No formal title	✓		1	✓	✓	GNLGAMS parallel stream [24]	1 year ROS/1 year of training [33]	Not specified
		✓	✓	2			GNLGAMS competitive stream [24]		
PEI	No formal title	✓		1	✓	✓	GNLGAMS parallel stream [24]	1 year ROS/1 year of training [33]	Not specified
		✓	✓	2			GNLGAMS competitive stream [24]		

*ROS* return of service; *CMG* Canadian medical graduates; *IMG* international medical graduates; *LCME/CACMS* Liaison Committee on Medical Education/Committee on Accreditation of Canadian Medical Schools

*Round—denotes the first or second iteration of the residency matching process

**Parallel—IMGs apply to a separate stream of positions than CMGs in one or more disciplines; competitive—IMGs apply to the same positions as CMGs in all disciplines

**Table 2:** Voluntary post-graduate medical education ROS programs for CMG* in Canada

Province	Program name	Program		Eligibility criteria	Funding	Time requirement	Payback amount
		Family Medicine	Other				
BC	Return of Service Program [22]	✓		Parallel stream for designated residency positions [23]	Not specified	1 year ROS/1 year training; to maximum of 3 years [22]	Training cost + interest + estimated damages (cost of replacement physician + average annual MSP billings). [22]
SK	Family Medicine Resident Bursary [39, 40]	✓		Specific for family medicine residents [40]	$25,000 per year (up to three years) [40]	1 year ROS/2 years in regional community, 1 year in rural community, or 6 months with SK Medical Association locum program [40]	All or a portion of the costs + interest [40]
MB	Northern Remote Family Medicine Stream (NRFMS) [41]	✓		Parallel stream**; required for anyone who is matched to this particular stream [28]	$50,000 taxable [28, 41]	2 years [28]	$50,000 + interest; can be prorated [28, 41]
	Medical Student/Resident Financial Assistance Program (MSRFAP) [38, 42]	✓	✓	Available to family medicine residents at the University of MB or a MB College of Medicine residency program in another Canadian/US university [42]	$20,000 grant [42]	1 year of ROS/1 year grant received [42]	Not specified
NL	Medical Resident Bursary Program [32]	✓	✓	Parallel and competitive streams; existing service need must be identified by the RHA [23]	$25–90,000 based on ROS community [32]	36 months/bursary [32]	Prorated cost of training based on number months of ROS unfulfilled + interest [43]
NS	Family Medicine Bursary [44]	✓		Available to full-time, part-time and locums [44]	$60,000 [44]	3 years [44–45]	Not specified
NB	Recruitment Incentives for New Physicians and Medical Residents (urban) [35]	✓		Available to residents in their last 2 years of residency training as well as new physicians, up to 6 months prior to their start date [35]	$30,000 [35]	2 consecutive years [35]	Not specified
	Recruitment Incentives for New Physicians and Medical Residents (rural) [35]	✓			$80,000 [35]	4 consecutive years [35]	
PEI	Family Medicine Physician Return-in-Service Grant [36]	✓		Physicians must be relocating from out of province or be in the PEI Family Medicine Residency Training Program and not have another ROS commitment [36]	Up to $90,000 (depending on location) [36]	Not specified	Repayment of funds + interest [36]
	Family Medicine Sponsorship Program [37]	✓			$80,000 [37]	5 years [37]	

*ROS* return of service; *CMG* Canadian medical graduates

*Voluntary ROS programs are available only to CMG

**Parallel—international medical graduates (IMG) apply to a separate stream of positions than CMG in one or more disciplines; competitive—IMG apply to the same positions as CMGs in all disciplines

How do the limited choice of available residency disciplines and ROS requirements shape the early-career decisions of IMGs in Canada? Using qualitative interviews, we examined resident and early-career IMG family physicians’ perceptions of the residency matching process and ROS requirements to understand how these two factors shaped their career decisions. For many IMGs, obtaining a residency position is viewed as the end of a long, challenging road to entering the physician workforce in Canada. While a number of studies have explored the experiences of IMGs as they qualify for residency programs [9, 10], few studies have examined IMGs as they enter practice in Canada or considered how residency conditions influence their career decisions. Given the role that IMGs play in addressing physician shortages in many countries, this study highlights the early-career workforce impacts of Canada’s policy approach.

## Methods

This study is a planned sub-analysis from a larger project examining early career influences of family physicians in Canada [11]. For the larger project, we conducted semi-structured qualitative interviews with early-career family physicians and family medicine residents in the Canadian provinces of British Columbia, Ontario, and Nova Scotia. “Early-career” is defined as family physicians who had been in practice for 10 years or less. We purposefully recruited participants along a wide range of personal and practice characteristics (i.e., maximum variation sampling [12]): gender, relationship status, parental status, practice setting, specialized training, and IMG versus CMG status. To recruit participants, study invitations were sent to current trainees and recent alumni of family medicine residency programs in British Columbia, Ontario, and Nova Scotia. We also recruited participants using social media (e.g., Twitter, Facebook), posters at research conferences, and notices at public talks given by study investigators. Additionally, Doctors Nova Scotia (the physicians’ union) emailed study invitations to their members.

Respondents to our study invitation initially completed an online screening survey using Opinio (ObjectPlanet Inc., Oslo, Norway), enabling us to assess for eligibility and gather demographic and practice-related information to facilitate purposeful sampling. We sent invitations to selected candidates and monitored survey responses on a weekly basis, which allowed us to adjust subsequent candidate selection, until we reached saturation (i.e., obtained sufficient data to allow for rigorous interpretation) in each province [12–14]. Participants were offered an honorarium.

### Data collection

A single researcher in each province conducted interviews with participants by phone or video-conference. At the start of each interview, the researcher obtained the participant’s verbal consent. The interviewer asked about the participant’s actual or intended practice characteristics (e.g., practice location, payment model) and the factors that shaped actual or intended practice, such as personal influences (e.g., family, gender, financial considerations), professional influences (e.g., professional satisfaction, work-life balance), experience during training (e.g., location, mentorship), and the policy environment (e.g., feasibility of practice). Different interview guides were used depending on whether the participant was a resident or early-career family physician. Interviews were recorded and transcribed verbatim. After each interview, the interviewer wrote field-notes to summarize key and/or unusual observations.

### Data analysis

The interview data were analyzed using an iterative multi-stage approach to thematic analysis [15]. Working with resident interviews, six members of the research team independently examined a single transcript line-by-line, using an inductive approach to identify a preliminary set of key themes [15]. The research team discussed these initial themes and then compared them to factors previously identified in the literature to create a preliminary coding template. The research assistants applied these preliminary codes to three additional resident transcripts and identified additional codes, which were then discussed by a working group of researchers and clinician-partners. The working group refined the coding template, which was then tested on a separate single transcript by ten team members, leading to a final version of the coding template. The three research assistants used the final template to code the full set of resident transcripts, using NVivo version 12.0 (software designed to assist in the organization and management of qualitative data). We integrated additional emergent codes into the coding template and applied them retroactively to any interviews that had already been coded.

We applied the codebook from the resident interviews to a sub-sample of physician interview transcripts, with 11 team members each coding the same physician transcript line-by-line. The resident coding template was then amended to generate a draft physician coding template. Three research assistants used the physician coding template to each code five interviews (*n* = 15) and another round of changes and additions were integrated into the template. Research assistants used this final physician coding template to code the physician interview transcripts using software described above (NVivo version 12.0). To ensure the consistency of the code book, each of the three research assistants coded one transcript from another province. For the sub-analysis portion of the study presented in this paper, we focused exclusively on interview data from IMGs and examined node reports for the codes IMG and ROS, and summarized recurring themes and identified illustrative quotes.

To protect confidentiality, we used study numbers to identify participants and have edited (demarcated by square brackets) potentially identifying information in quotations. For each quotation, we have also identified whether the participant was a resident (R) or physician (P), and the abbreviation of province in which they worked or trained (BC—British Columbia, ON—Ontario, NS—Nova Scotia).

### Positionality

The authors include individuals who are involved (directly or indirectly) in the selection and training of family medicine residents in Canada, family physicians in urban and rural locations, IMGs, and immigrants to Canada. Authors in the group held a range of personal views on ROS programs. Through discussion of drafts of the manuscript, we arrived at a description and interpretation of findings that balanced our individual views and reflected the data (quotations) gathered from study participants.

## Results

Across the three provinces, 31 of 32 invited residents and 63 of 65 invited early career physicians completed an interview. One invited resident and one invited physician did not participate due to scheduling conflicts, and one invited physician withdrew without providing a reason. Interviews were between 45 and 60 minutes in length.

For this sub-analysis, which focussed on IMG-specific issues, we included only IMG study participants who comprise 9 (37.5%) residents and 15 (62.5%) physicians (Table 3). The majority of IMG participants were female (54.2%), partnered (70.8%), and had children (58.3%). Twelve of the 16 physicians had been in practice for less than 5 years (four had been in practice for less than 2 years and still would have been serving their ROS commitment, if applicable). Many of the participants were Canadians who studied abroad [1], that is, trainees who were Canadian citizens or permanent residents before attending medical school abroad.

**Table 3:** Characteristics of international medical graduate study participants

Participant characteristics	*n* = 24 *n* (%)
**Career stage**	
Physician	15 (62.5)
Resident	9 (37.5)
Province*	
British Columbia	8 (33.3)
Ontario	10 (41.7)
Nova Scotia	7 (29.2)
Gender**	
Male	11 (45.8)
Female	13 (54.2)
**Relationship status**	
Partnered (married or equivalent)	17 (70.8)
Un-partnered (single or equivalent)	7 (29.2)
**Have children**	
No	10 (41.7)
Yes	14 (58.3)
**Years in practice**	
0 (still in residency)	9 (37.5)
1–5	12 (50.0)
6–10	3 (12.5)

*Adds to more than 100% because 1 participant worked in 2 provinces

**Participants were asked to report their gender in an open-text response; only these two categories were reported by participants

We identified three themes: IMGs strategically chose family medicine to increase the likelihood of obtaining a residency position; ROS limited career choices; and ROS agreements delayed preferred practice choices for IMGs.

### Family medicine as a strategic residency choice

The limited availability of IMG residency training options in Canada had a profound influence on study participants’ training choices. While some were drawn to family medicine for other reasons, most indicated they had opted to specialize in family medicine to increase their likelihood of matching (i.e., obtaining a residency position in Canada). For a number of IMGs, for example, positive experiences during medical school electives and strong mentors were very influential in their choice of family medicine: “*And one of the doctors that I worked with was amazing. And she kind of made me excited about doing family medicine”* (P62 BC), but the residency match was always an overriding concern:

*I think I had some very good mentors in primary care, in family medicine in terms of like seeing other bright, like young engaged female physicians that were really enjoying family practice. And that was a pretty big influence on me. And then realistically the other biggest influence was the CaRMS [Canadian Resident Matching Service] matching process, and the number of spots for family medicine versus for other specialties. And so, when you’re an international graduate, you’re playing a bit of a numbers game to get back into Canada. And so that probably influenced me as much as anything else did* (P20 BC).

Other participants noted that they likely would have chosen family medicine even if other options offered the same likelihood of being able to train in Canada. For example, a physician now practicing in Nova Scotia recalled that:

*I did a number of my family medicine rotations in med school back in Canada. And I did them mostly in Ontario but with a few really great family physicians who had really great practices. And I think that helped influence me. And then to be completely honest, I didn’t want to stay [abroad]. I wanted to come home [to Canada]. And trying to get an IMG residency spot in anything other than family medicine, I think… I didn’t even think I’d get a family med one. But that was a big factor though as well. Like you know, I wanted to be back here, and I wanted to do my residency here. And family medicine provided the most opportunities for me. But I’m glad I chose it. I’m glad I’m not a radiation oncologist* (P22 NS).

However, for others, family medicine was chosen over other specialties as a trade-off to increase their chances in the match: “*For a while I thought maybe I’d like to do neurology or something. But I feel like anything that would have been competitive would have been really tough for me to get as an IMG grad rather than as a Canadian grad. So that was probably the biggest influence*” (P51 ON).

### ROS restricted career choices

For many IMGs, the ROS requirement was an unwelcome feature of the post-graduate medical system in Canada, and many felt that they had no real choice about accepting a ROS agreement: “*One can say that we signed the return of service contract willfully, and we could choose not to sign it. But I want this to be recorded if possible that it was not a choice…they [IMG] are doing it because they have no other option*…” (R8 BC). Study participants indicated the ROS also restricted their choices of subsequent training and future type of practice. For example, when asked whether he was considering completing a supplementary third year of focused training (i.e., PGY3), an IMG physician believed that: “*You are not allowed to do anything else until you have completed your return of service. So, you can do it afterwards. But then you’re 3 years out in practice”* (P20 BC). He believed that while he could apply to complete the additional training after the service commitment, he was unlikely to be competitive in the PGY3 match, because he felt his years out of residency would disadvantage him when competing against applicants currently in residency.

### ROS delays preferred practice choices

ROS requirements also created much uncertainty for IMGs. Most IMGs do not know, where they will be completing their ROS or the nature of their practice during their ROS until late in their residency training. When asked about future practice intentions, residents responded: “*I’m unable to comment on that at this time. And the reason is that because I do not yet know the details of my return of service. That will occur over the next year or so. But at this point I can't answer that question”* (R15 NS) and “*I’m not sure because I don't know where my return of service agreement will be. I don’t necessarily know if I’ll have the flexibility to create the practice that I’d ultimately like to have my career into”* (R14 NS).

It is not always possible for an IMG to obtain a ROS agreement that allows them to practice clinically in their preferred way. For example, an IMG in Ontario noted that she may have little choice about the scope of practice of her work given that her choices are dictated by Ministry regulations:

*And I guess whether I end up working, doing hospital work or not, in the next few years is probably because the Ministry is making it a regulation for us or forcing us to do it for the FHO [Family Health Organization, a model of primary care]. It’s hard to say whether I would agree to that or not. But yeah, I sort of am at the mercy of some of the regulations* (P51 ON).

Another IMG physician in Ontario was able to find a position that met ROS requirements and allowed her to work in a hospital, but because she was required to work in a smaller community, the ROS requirements prevented her from more longer term considerations, such as setting up an independent practice:

*I did not think about opening my own practice independently because my return of service requires me to work outside of the [city name], outside of [another, larger metropolitan area]. … I found I really enjoy hospital work. So I decided I would do hospitalist. I was directed to. I had to be outside of the city limits for full-time work* (P47 ON).

Similarly, a physician in British Columbia noted that the nature of her clinical work, as well as her location, would likely change once she was free to make her own choices: “*Like probably I will change the practice format that I’m doing once my return of service contract is over. I may or may not stay in the current practice I’m in. Yeah, that’s yet to be decided. But it’s probably about right now 50/50 whether I would stay or not”* (R8 BC).

For a few residents, the ROS requirements aligned with their longer term intentions and allowed them to practice where and how they had wanted. For these physicians, the ROS requirements imposed minimal barriers to their ability to fulfill their intended practice. In Ontario, ROS agreements require IMGs to work outside the two large metropolitan areas. For an IMG resident who hoped to work in a specific city in Ontario outside of these two regions, the ROS agreement aligned with her intended plans: “*I don't think it has an effect on my practice because for the return of service, it's only that you can’t practice in certain areas in Ontario…. Because my goal is to work in [city name]*” (R30 ON). Similarly, ROS obligations did not interfere with plans for an IMG who had wanted to practice in a rural location: “*For me it's my interest in rural practice. And actually having the return of service was not a reason why I … like it didn’t matter to me coming back because … it wasn’t a barrier because that’s what I wanted*” (R3 BC). For one IMG, the ROS obligation facilitated her ability to work in a community, where she had family and where her spouse was employed: “*Like I guess I was fortunate that like the place I lived in and where my family is…like my husband was working, was…. So I was lucky I didn’t have to move or anything to fulfil my return of service obligations*” (P58 ON).

## Discussion

In this study, IMGs wanting to practice in Canada strategically chose family medicine to increase the likelihood that they would obtain a residency position. Few studies have examined the impact of physicians who were unable to train in the speciality of their choice. However, a survey of CMGs whose match to family medicine was not their first choice found that respondents viewed family medicine as a viable career option. While they assigned less value to their own status as a family physician and to family medicine as a discipline at the end of their residency training than physicians who matched to family medicine as their first choice, they nonetheless felt well-prepared to practice and had favourable impressions of the lifestyle that family medicine provided [16].

ROS agreements influenced participants’ early-career practice intentions by restricting training choices and delaying preferred scope of practice and work in preferred locations. Many IMGs described their initial practice under the ROS requirement as an acquiescence to government regulations. It is unclear how ROS agreements affect the practice of IMGs in the longer term, once the service obligations have been completed. While a recent national survey of family medicine residents in Canada found that IMGs are more likely than CMGs to intend to provide comprehensive, longitudinal patient care within the first 3 years of practice, the survey did not distinguish between respondents with and without ROS obligations [17]. It is therefore unclear whether the responses in this national survey reflect IMGs’ ROS practice intentions or their practice intentions in the longer term, after their ROS commitment.

Many countries impose practice and location restrictions on IMGs and foreign students in domestic medical schools [4]. Understanding the training and regulatory contexts in which these policies are used is integral to evaluating their impacts. Canada’s approach of requiring IMGs to complete ROS agreements may contribute to high turnover (and an unstable rural workforce) if ROS opportunities are not aligned with IMGs’ location and scope of practice preferences. We found that although IMG study participants were directed to complete their ROS obligation outside large urban centers, few suggested that they intended to remain in these smaller communities in the long term. Evaluations of ROS agreements in Canada have produced mixed results, and highlight that the contract terms and the regulatory and licensing context are important considerations. For example, an evaluation of residency-linked ROS agreements found that IMGs accounted for almost three-quarters of trainees who defaulted (neither fulfilled service nor repaid funding) on their agreement, but the overall number of IMGs in the study was small [18]. In contrast, a recent evaluation of IMG ROS agreements in Manitoba found that roughly 60% of IMGs remained in rural communities after the service obligation was complete [17]. However, these IMGs did not complete residency training in Canada and had not obtained licensing credentials that would allow them to move to other provinces, which may have curtailed their options. Future studies, in Canada and elsewhere, should examine ROS fulfillment rates, the key contextual factors associated with ROS completion, and the impact of ROS agreements on IMG work location and practice patterns after ROS obligations are fulfilled.

While workforce analysts call for policies in destination countries such as Canada to support the WHO Global Code of Practice in the International Recruitment of Health Personnel [19, 20], the IMGs included in the study are unlikely to have been actively recruited to immigrate to Canada and fill physician shortages. IMGs who are actively recruited to Canada are able to practice with provisional or restricted licenses without having completed residency training [17, 21]. Rather, ROS requirements illustrate the conflict between policies that encourage the immigration of highly educated professionals and health workforce policies that inhibit immigrants’ ability to work in their profession in Canada.

### Limitations

Our study only included data from IMG family physicians and included neither IMGs who matched to other specialist residency programs, nor IMGs who did not match to a residency position. While our participants represented a range of provinces and genders, further research is needed to examine the perspectives of IMGs excluded from the study, because they were unable to obtain a residency position and/or qualify for practice in Canada. Our participants were IMGs who trained or worked in family medicine in British Columbia, Nova Scotia, and Ontario; the results of the study may not be transferable to other groups of specialists, CMGs, or physicians who worked and trained in other provinces of Canada. Participants may also have been influenced by social desirability bias, that is, providing answers that they believe will be viewed more favourably, such as their attitudes towards providing comprehensive care or their intention to fulfil ROS obligations. To mitigate this, participants were assured anonymity and the interviewers were trained to remain neutral in their responses and questions. Finally, our interpretation of the data may be influenced by our own personal views and biases. To address this limitation, all authors reviewed a draft of the manuscript to ensure the description and interpretation of findings were driven by study data.

## Conclusions

The residency match is a critical period in the careers of medical trainees in Canada. ROS agreements are obligatory for most IMGs who complete residency training in Canada. Many IMGs strategically choose to specialize in family medicine to increase the likelihood of being able to work in Canada. Our study found that ROS agreements can restrict career choices and delay IMGs from their preferred scope of practice and location. Since most existing analyses of IMG early-career practice do not distinguish between ROS and self-determined practice, further research is needed to understand how ROS requirements influence the longer term practice patterns of IMGs.

## Data Availability

The data sets generated and/or analysed during the current study are not publicly available due participant confidentiality but are available from the corresponding author on reasonable request.

## Abbreviations

IMG: International medical graduate
ROS: Return of service
CMG: Canadian medical graduate
NS: Nova Scotia
BC: British Columbia
ON: Ontario
CaRMS: Canadian Resident Matching Service
FHO: Family Health Organization

## References

[1] Thomson G, Cohn K. IMG selection: Independent review of access to postgraduate programs by international medical graduates in Ontario. Volume 1: Findings and recommendations. Ontario Ministry of Health and Long-Term Care and the Council of Ontario Universities. 2011. http://www.health.gov.on.ca/en/common/ministry/publications/reports/thomson/v1_thomson.pdf. Accessed 21 Sept 2021.

[2] Canadian Residency Matching Service. 2021 CaRMS Forum. CaRMs. 2021. https://www.carms.ca/data-reports/r1-data-reports/ Accessed 21 Sept 2021.

[3] Canadian Residency Matching Service. Eligibility criteria. CaRMs. 2021. https://www.carms.ca/match/r-1-main-residency-match/eligibility-criteria/. Accessed 04 Aug 2021.

[4] Bärnighausen T, Bloom DE (2009). Financial incentives for return of service in underserved areas: a systematic review. BMC Health Serv Res.

[5] Mason HR (1971). Effectiveness of student aid programs tied to a service commitment. J Med Educ.

[6] Neufeld SM, Mathews M (2011). Canadian return for service bursary programs for medical trainees. Healthc Policy.

[7] Pathman DE, Taylor DH, Konrad TR, King TS, Harris T, Henderson TM (2000). State scholarship, loan forgiveness and related programs: The unheralded safety net. JAMA.

[8] Sempowski IP (2004). Effectiveness of financial incentives in exchange for rural and underserviced area return-of-service commitments: Systematic review of the literature. Can J Rural Med.

[9] Neiterman E, Bourgeault IL (2015). Professional integration as a process of professional resocialization: internationally educated health professionals in Canada. Soc Sci Med.

[10] Neiterman E, Bourgeault IL, Covell C (2017). What do we know and not know about the professional integration of international medical graduates (IMGs) in Canada? Recommendations for research and policy from a scoping review of the literature. Healthc Policy.

[11] Lavergne MR, Goldsmith LJ, Grudniewicz A, Rudoler D, Marshall EG, Ahuja M (2019). Practice patterns among early-career primary care (ECPC) physicians and workforce planning implications: protocol for a mixed methods study. BMJ Open.

[12] Patton MQ (2015). Qualitative research & evaluation methods.

[13] Morse J, Denzin NK, Lincoln YS (1994). Designing funded qualitative research. Handbook of qualitative research.

[14] Burke LA, Miller MK (2001). Phone interviewing as a means of data collection: Lessons learned and practical recommendations. Forum Qual Soc Res.

[15] Aronson J (1995). A pragmatic view of thematic analysis. Quality.

[16] Woloschuk W, Myhre D, Dickinson J, Ross S (2017). Implications of not matching to a first-choice discipline: a family medicine perspective. Can Med Educ J.

[17] Mowat S, Reslerova M, Sisler J (2017). Retention in a 10-year cohort of internationally trained family physicians licensed in Manitoba. Can J Rural Med.

[18] Mathews M, Heath SL, Neufeld SM, Samarasena A (2013). Evaluation of physician return-for-service agreements in Newfoundland and Labrador. Healthc Policy.

[19] Bourgeault IL, Labonté R, Packer C, Runnels V, Murphy GT (2016). Knowledge and potential impact of the WHO global code of practice on the international recruitment of health personnel: Does it matter for source and destination country stakeholders?. Hum Resour Health.

[20] Tam V, Edge JS, Hoffman SJ (2016). Empirically evaluating the WHO global code of practice in the international recruitment of health personnel’s impact on four high-income countries four years after adoption. Glob Health.

[21] Mathews M, Edwards AC, Rourke JTB (2008). Retention of provisionally licensed international medical graduates: a historical cohort study of general and family physicians in Newfoundland and Labrador. Open Med.

[22] British Columbia Ministry of Health. Return of service program policy- August 2019. BC Ministry of Health Human Resources and Labour Relations Division. 2019. https://www2.gov.bc.ca/assets/gov/health/practitioner-pro/ros-policy-2020.pdf. Accessed 22 Sept 2021.

[23] Canadian Resident Matching Service. Summary of intake criteria for international medical graduates (IMGs) by province – 2021 cycle information. CaRMs. 2020. https://www.carms.ca/match/r-1-main-residency-match/eligibility-criteria/summary-intake-criteria-international-medical-graduates-imgs-province/. Accessed 22 Sept 2021.

[24] Canadian Resident Matching Service. Provincial criteria. CaRMS. 2021. https://www.carms.ca/match/r-1-main-residency-match/eligibility-criteria/. Accessed 23 Sept 2021.

[25] British Columbia Medial Association. Practice In BC. Doctors of BC. 2016. https://practiceinbc.ca/practice-in-bc/img-au-irl-uk-usa-residency-ca/return-of-service. Accessed 22 Sept 2021.

[26] Saskatchewan Health Authority (Saskdocs). Return of service (ROS) questions and answers. Saskdocs. n.d. https://www.saskdocs.ca/work/ros/. Accessed 22 Sept 2021.

[27] Saskatchewan Health Authority (Saskdocs). IMG postgraduate medical training agreement. Saskdocs. n.d. https://medicine.usask.ca/documents/pgme/international-medical-graduate-img-agreement_2020.pdf. Accessed 22 Sept 2021.

[28] University of Manitoba. CaRMs application FAQ. Department of Family Medicine. 2020. https://umanitoba.ca/faculties/health_sciences/medicine/units/family_medicine/orientation/carms-faq.html. Accessed 23 Sept 2021.

[29] Ontario Ministry of Health and Long-Term Care. Physician return of service (ROS) programs. Health Workforce Planning Branch. 2019. https://www.health.gov.on.ca/en/pro/programs/hhrsd/physicians/ros.aspx. Accessed 23 Sept 2021.

[30] Ontario Ministry of Health and Long-Term Care. International medical graduate return of service program guidelines. Government of Ontario. n.d. https://www.health.gov.on.ca/en/pro/programs/hhrsd/physicians/docs/IMG_ROS_Guide.pdf. Accessed 23 Sept 2021.

[31] Practice Newfoundland Labrador. Bursary/fellowship opportunities. Government of NL. 2021. https://www.practicenl.ca/jobs/content/bursaries.asp#family. Accessed 23 Sept 2021.

[32] Department of Health and Community Services. Bursaries/incentives. Government of NL. 2021. https://www.gov.nl.ca/hcs/grantsfunding/bursaries/. Accessed 23 Sept 2021.

[33] Canadian Resident Matching Service. Nova Scotia, New Brunswick, and Prince Edward Island – 2021 cycle information. CaRMS. 2021. https://www.carms.ca/match/r-1-main-residency-match/eligibility-criteria/nova-scotia-new-brunswick-pei/#1594752203091-ee3145f0-2e09. Accessed 22 Sept 2021.

[34] Dalhousie University. Postgraduate medical education: Return of service agreement. Dalhousie University. 2021. https://medicine.dal.ca/departments/core-units/postgraduate/admissions/ros_agreement.html. Accessed 22 Sept 2021.

[35] Government of New Brunswick. Recruitment incentives for new physicians and medical residents: 2021–2022 program guidelines. Government of NB. 2021. https://www2.gnb.ca/content/gnb/en/corporate/promo/careers_in_healthcare/Recruitment-and-Retention-Incentives.html. Accessed 22 Sept 2021.

[36] Government of Prince Edward Island. Financial incentives for physicians – effective January 2019. Department of Health and Wellness. 2021. https://www.princeedwardisland.ca/en/information/health-and-wellness/financial-incentives-physicians. Accessed 22 Sept 2021.

[37] Government of Prince Edward Island. Family medicine sponsorship program. Department of Health and Wellness. 2021 https://www.princeedwardisland.ca/en/information/health-and-wellness/family-medicine-sponsorship-program. Accessed 22 Sept 2021.

[38] Government of Manitoba. Medical student/resident financial assistance program (MSRFAP). Government of Manitoba. n.d. https://www.gov.mb.ca/health/msrfap/. Accessed 22 Sept 2021.

[39] Saskatchewan Medical Association. Bursaries: Family medicine resident bursary. SMA. 2020. https://www.sma.sk.ca/programs/27/bursaries.html. Accessed 22 Sept 2021.

[40] Saskatchewan Health Authority (Saskdocs). Return-of-service policies and guidelines. Saskdocs. 2018. https://www.saskdocs.ca/web_files/ReturnOfServicePolicy_Jan2018.pdf. Accessed 22 Sept 2021.

[41] University of Manitoba. Northern stream. Department of Family Medicine. 2020. https://umanitoba.ca/faculties/health_sciences/medicine/units/family_medicine/streams/northern.html. Accessed 23 Sept 2021.

[42] Government of Manitoba. Medical student/resident financial assistance program (MSRFAP). Government of Manitoba. 2016. http://digitalcollection.gov.mb.ca/awweb/pdfopener?smd=1&did=25023&md=1. Accessed 22 Sept 2021.

[43] Department of Health and Community Services. Provincial physician bursary program policy. Government of NL. 2016. https://www.gov.nl.ca/hcs/files/grantsfunding-pdf-physician-bursary-program-policy.pdf. Accessed 22 Sept 2021.

[44] Government of Nova Scotia. News release- physician incentive program changes add flexibility, choice. Province of Nova Scotia. 2018. https://novascotia.ca/news/release/?id=20180417002. Accessed 22 Sept 2021.

[45] Doctors Nova Scotia. Return of service arrangements. Doctors Nova Scotia. n.d. https://doctorsns.com/contract-and-support/ros. Accessed 22 Sept 2021.

